# Polymyxin B Nephrotoxicity: From Organ to Cell Damage

**DOI:** 10.1371/journal.pone.0161057

**Published:** 2016-08-17

**Authors:** Maria de Fátima Fernandes Vattimo, Mirian Watanabe, Cassiane Dezoti da Fonseca, Luciana Barros de Moura Neiva, Edson Andrade Pessoa, Fernanda Teixeira Borges

**Affiliations:** 1 Experimental Laboratory of Animal Models (LEMA), School of Nursing of the University of Sao Paulo, Sao Paulo, Brazil; 2 Nephrology Division, Federal University of Sao Paulo, Sao Paulo, Brazil; Centro Cardiologico Monzino, ITALY

## Abstract

Polymyxins have a long history of dose-limiting toxicity, but the underlying mechanism of polymyxin B-induced nephrotoxicity is unclear. This study investigated the link between the nephrotoxic effects of polymyxin B on renal metabolic functions and mitochondrial morphology in rats and on the structural integrity of LLC-PK1 cells. Fifteen Wistar rats were divided into two groups: Saline group, rats received 3 mL/kg of 0.9% NaCl intraperitoneally (i.p.) once a day for 5 days; Polymyxin B group, rats received 4 mg/kg/day of polymyxin B i.p. once a day for 5 days. Renal function, renal hemodynamics, oxidative stress, mitochondrial injury and histological characteristics were assessed. Cell membrane damage was evaluated via lactate dehydrogenase and nitric oxide levels, cell viability, and apoptosis in cells exposed to 12.5 μM, 75 μM and 375 μM polymyxin B. Polymyxin B was immunolocated using Lissamine rhodamine-polymyxin B in LLC-PK1 cells. Polymyxin B administration in rats reduced creatinine clearance and increased renal vascular resistance and oxidative damage. Mitochondrial damage was confirmed by electron microscopy and cytosolic localization of cytochrome c. Histological analysis revealed tubular dilatation and necrosis in the renal cortex. The reduction in cell viability and the increase in apoptosis, lactate dehydrogenase levels and nitric oxide levels confirmed the cytotoxicity of polymyxin B. The incubation of LLC-PK1 cells resulted in mitochondrial localization of polymyxin B. This study demonstrates that polymyxin B nephrotoxicity is characterized by mitochondrial dysfunction and free radical generation in both LLC-PK1 cells and rat kidneys. These data also provide support for clinical studies on the side effects of polymyxin B.

## Introduction

Polymyxins were discovered late in 1947, but their toxicity has limited their use. Recently, polymyxins have been reintroduced to clinical practice due to the prevalence of infections caused by gram-negative bacteria such as multidrug-resistant *Pseudomonas aeruginosa*, *Acinetobacter baumannii* and *Klebsiella pneumoniae* [1, 2, 3]. However, systemic polymyxin therapy is associated with a high incidence of acute kidney injury (AKI). Nephrotoxicity is the major dose-limiting factor restricting its use in clinical practice, occurring in up to 60% of all patients treated with polymyxins [4, 5, 6].

Most of the drugs that cause nephrotoxicity exert their toxic effects via one or more of the following mechanisms: renal hypoperfusion, tubular cell toxicity, inflammation and oxidative stress [7]. The elucidation of the mechanisms of action, the concentration-dependent capacity to kill bacterial, and the dose-limiting adverse effects of polymyxins would contribute to the management of their use in patients.

Polymyxins are cyclic lipopeptide antibiotics, but only polymyxins B and E (colistin) are available for clinical use. The potent direct nephrotoxic effects of polymyxins include mechanisms that kill bacteria via interactions with lipid A, disrupting the Ca^2+^ and Mg^2+^ bridges, which destabilizes the lipopolysaccharide molecules in the bacterial membrane. [3, 8]. Therefore, the nephrotoxicity of polymyxins appears to be due to effects on D-amino content and fatty acid components that increase membrane permeability and the influx of cations [9].

The results of our study appear to confirm previous data showing the mitochondrial accumulation of polymyxin B [10], resulting in mitochondrial dysfunction in LLC-PK1 cells, a model of porcine tubular cells, via free radical production. These findings were also corroborated by our animal model results.

## Materials and Methods

### Chemicals and Reagents

The following cells and chemicals were purchased from the indicated suppliers: immortalized LLC-PK1 cells_,_ ATCC-American Type Culture Collection, USA; polymyxin B, Bedford Laboratories, USA; fetal bovine serum, Gibco, USA; Dulbecco’s Modified Eagle’s Medium (DMEM), Sigma, USA; acridine orange/ethidium bromide, Sigma, USA; HOE 33342 [bisbenzimide (2'-[4-ethoxyphenyl]-5-[4-methyl-1-piperazinyl]-2,5'-bi-1H-benzimidazole) trihydrochloride] dye, Sigma, USA; xylenol orange [o-cresolsulfonphthalein-3′,3″-bis(methylimino) diacetic acid], Sigma, USA; DTPA (diethylenetriamine-N,N,N*′*,N*″*,N*″-*pentaacetate), Sigma, USA; DTNB [5,5′-dithiobis(2-nitrobenzoic acid)], Sigma, USA; and naphthylethylenediamine, Sigma, USA.

### In vivo studies

The in vivo studies were approved by the Ethics Committee for the Use of Experimental Animals, University of Sao Paulo (CEEA–protocol n. 038/08) and were performed in accordance with international standards for the manipulation and care of laboratory animals. Adult male Wistar rats were housed in a room at a controlled temperature (25°C/77°F) on alternating light/dark cycles and had free access to water and rat chow (Nuvilab CR-1, Nuvital, Brazil).

#### Animal grouping

Male Wistar rats weighing 286 ± 12 g were provided by the University of Sao Paulo—School of Medicine animal facility. A total of 15 rats were divided into two groups: (i) Saline control group (n = 7): rats received 3 mL/kg 0.9% NaCl intraperitoneally (i.p.) once a day for 5 days; (ii) Polymyxin B group (n = 8): rats received 4 mg/kg/day polymyxin B i.p. once a day for 5 days [11,12].

#### Procedures and timing

Metabolic cages were used for the collection of urine samples. On the fifth day, immediately after the last injection, rats were placed in metabolic cages for a 24-hour measurement of urinary volume and the collection of urine samples.

#### Mean arterial pressure and renal blood flow measurements

Following the 24-hour period in the metabolic cage, animals were anesthetized with 50 mg/kg body weight (BW) of sodium thiopental and a polyethylene tube (PE-50) was inserted into the right carotid artery to monitor mean arterial pressure (MAP) and heart rate (HR) using an electronic transducer connected to a data acquisition program (TSD104A, BIOPAC Systems Inc., USA). Then, a ventral midline incision was made, exposing the left renal pedicle. The renal artery was then isolated, and a suitable probe was placed around the exposed artery to measure renal blood flow (RBF) [13].

#### Collection of blood samples

Blood samples were collected by puncturing the abdominal aorta. Animals were euthanized at the end of the experiment according to the guidelines for animal experimentation [14].

#### Tissue sample collection/preparation

The left kidney was prepared for electron microscopic examination and histological analysis.

#### Renal function

Serum and creatinine concentrations in the urine were measured using the Jaffe method, and creatinine clearance was calculated based on the following formula: creatinine clearance = [urine creatinine concentration (mg/dL) × 24-hour urinary volume (mL/min)] / serum creatinine concentration (mg/dL). The calculated creatinine clearance rate (mL/min) was normalized to the weight of the rat [15].

#### Renal hemodynamics

RBF measurements were made using an ultrasonic flowmeter (T402, Transonic Systems Inc., USA). Renal vascular resistance (RVR) was calculated based on the standard formula: RVR = MAP / RBF [13].

#### Oxidative stress

(i) Urinary peroxides were determined based on version 2 of the ferrous oxidation of xylenol orange (FOX-2) method. Xylenol orange shows a high selectivity for Fe^3+^, producing a bluish purple complex (A = 4.3 × 10^4^ M^-1^ cm^-1^). The values were corrected for urinary creatinine content (in grams) and are expressed as nmol/g creatinine [16]. (ii) Non-protein soluble thiols in renal tissue were assessed in tissue homogenized in 1 mL of a solution containing 10 mM sodium acetate, 0.5% Tween 20 and 100 μM DTPA (pH 6.5). One aliquot was reserved for the immediate measurement of total protein content, and the second aliquot was precipitated with 20% trichloroacetic acid (1:1) to measure total thiol content. Deproteinized samples were homogenized in 100 μl of a solution containing 100 mM Tris buffer (pH 8.0). After 10 min at room temperature, the quantity of thiols was determined based on the mean absorbance at 412 nm (A = 13.6 x 10^3^ M^-1^ cm^-1^). The amount of soluble thiols was corrected for the total protein content and is expressed as nmol/mg total protein [17]. (iii) Catalase activity was measured following the homogenization of renal tissue in phosphate-buffered saline (PBS). One aliquot was used for the measurement of total protein content. The other aliquot was mixed with 1 M Tris-HCl, 50 mM ethylenediaminetetraacetic acid (EDTA) and 10 mM H_2_O_2_, and its absorbance was read in a spectrophotometer at 240 nm for 2 min at room temperature. The results are reported as the decrease in nmol of H_2_O_2_ per min/mg total protein [18]. (iv) Lipid peroxidation levels of malondialdehyde were determined by measuring thiobarbituric acid-reactive substances (TBARS). For peroxidation quantification, 0.4 mL of a urine sample mixed with 0.6 mL water was added to a reaction mixture consisting of 1.0 mL 17.5% trichloroacetic acid (TCA) and 1.0 mL 0.6% thiobarbituric acid. This mixture was heated in a water bath at 95°C for 20 min, and the solution was removed from the water bath, cooled on ice, and mixed with 1.0 mL 70% TCA. The solution was then homogenized and incubated for 20 min. Finally, the solution was read in a spectrophotometer at 535 nm (A = 1.56 × 10^5^ M^-1^ cm^-1^. The values are expressed as nmol/g creatinine [19].

#### Electron microscopy examination

Animals were perfusion-fixed through the renal artery with 100 mL PBS and a buffered 2% formaldehyde/2.5% glutaraldehyde solution. Slices of the left kidneys were removed and placed in the same fixative at room temperature for 30 minutes. They were then kept cool until processing. Briefly, samples were washed with 0.1 M sodium cacodylate buffer (pH 7.2), post-fixed in 2% osmium tetroxide, dehydrated in ethanol, cleared with propylene oxide, and infiltrated with a mixture of propylene oxide/EPON resin in increasing concentrations. The samples were embedded in pure EPON resin for polymerization at 60°C for 48 hours. After curing, the blocks were trimmed to obtain semi-thin slices for the identification of glomeruli under a light microscope. Then, blocks were trimmed again to obtain ultrathin sections, which were deposited on fenestrated metal gratings for a final staining with uranyl acetate and lead citrate. Samples were analyzed using a 1200 EXII transmission electron microscope (JEOL, Japan) coupled to a Gatan Orius digital camera system (USA). [20]

### Western blot detection and quantification of cytochrome c protein in mitochondrial and cytosolic fractions

Kidney cortices were cut (100 mg) and homogenized in the medium (5 mL/g of tissue) containing Sucrose 300 mM, EDTA 0.5 mM, 20mM Tris-HCl (pH 7.4), supplemented with a protease inhibitor cocktail (Roche Applied Science, Germany). After homogenization, cytosolic and mitochondrial fractions were separated by differential centrifugation (5 min at 1000xg, 10 min at 9000xg). Supernatant was centrifuged for 60 min at 100000xg and the resulting supernatant was used for immunoblotting. Total cytosolic and mitochondrial protein was measured by bicinchoninicc acid protein assay (Thermo Fisher Scientific, USA) [21].

#### Histological analysis

Slices of the left kidney were immersed in Bouin’s solution for 4 h. Subsequently, renal tissues were placed in a series of baths of 70% alcohol for the elimination of picric acid, dehydrated and embedded in paraffin. The paraffin sections of perfused/fixed kidneys were stained with hematoxylin and eosin for histological analysis via light microscopy. Tubulointerstitial damage was defined as tubular necrosis, the presence of an inflammatory cell infiltrate, tubular lumen dilatation or tubular atrophy [22].

### In vitro studies

A pig proximal tubular epithelial cell line (immortalized LLC-PK1 cells) obtained from ATCC were used from passage seven. Cells were maintained in culture flasks containing DMEM supplemented with 5% fetal bovine serum. Cells were grown to semiconfluence at 37°C in a humidified atmosphere containing 5% carbon dioxide. LLC-PK1 cells cultivated on multiwell plates (12 wells) were divided into the following groups: Control (n = 8), cells exposed to 12.5 μM polymyxin B (n = 8), cells exposed to 75 μM polymyxin B (n = 8) and cells exposed to 375 μM polymyxin B (n = 8).

#### Viability and apoptosis of LLC-PK1 cells in the presence of polymyxin B

(i) Cell viability was assessed using the acridine orange/ethidium bromide method. After the experimental treatment, DMEM culture media were collected and the cells were washed with Dulbecco’s PBS. After washing, the cells were trypsinized and centrifuged at 1200 rpm for 4 min. The cell precipitate was homogenized, and 10 μL of this suspension was mixed with 0.3 μL of a solution containing the dyes acridine orange and ethidium bromide (100 mg/L) at a ratio of 1:1 (v/v). Ethidium bromide passes through intact membranes, binding to cellular DNA and staining cells green, indicating viable cells. Acridine orange stains RNA but does not pass through intact membrane, so orange-red fluorescence indicates inviable cells. Cells were counted under a fluorescence microscope, and the results are reported as the percentage of viable cells at 24 and 72 hours. (ii) The evaluation of apoptosis was performed using the Hoechst 33342 staining method. LLC-PK1 cell suspensions were centrifuged, resuspended in PBS and incubated with 10 μL Hoechst 33342 (1.0 mM) solution for 5–15 min for chromatin staining [21]. Cells were observed under a light microscope. Blue cells with uncondensed chromatin were considered to be non-apoptotic, and cells with condensed chromatin were considered to be apoptotic. At least 100 cells per culture flask were counted, and the results are reported as the percentage of necrotic or apoptotic cells at 24 and 72 hours [23].

#### Mediators of membrane damage in LLC-PK1 cells in the presence of polymyxin B

(i) The release of the intracellular enzyme lactate dehydrogenase (LDH) was measured. Cells were scrapped from culturing flasks in PBS to obtain cell homogenates. Sodium pyruvate (1%), NADH (0.2 mM), Tris-HCl (0.1 M) and EDTA (0.5 mM) were added to the samples. Enzymatic activity was calculated by measuring the absorbance of the samples at 340 nm. (ii) Nitric oxide (NO) levels in the cell culture media were determined via the Griess method. Briefly, a solution of 1% sulfanilamide in 5% H_3_PO_4_ containing 0.1% naphthylethylenediamine was added to aliquots of media, and absorbance was measured at 546 nm. Nitrite, a stable metabolite of NO, was then quantified using a standard curve based on NaNO_2_ (sodium nitrite). The protein content of the culture media was assessed based on an albumin standard curve [24].

#### Assay of mitochondrial function in LLC-PK1 cells in the presence of polymyxin B

Fluorescent polymyxin B was used to assess mitochondrial function. Polymyxin B (43.2 μMoles, FW 1,301) was added to 22.8 reactive moles of Lissamine rhodamine B sulfonyl chloride (FW 577) at a conjugation efficiency of 33% and in a 2:1 stoichiometric ratio. This ratio was used to minimize the occurrence of double conjugates of polymyxin B while retaining a greater fluorescence/mg of compound. The incubation in the media lasted 6 hours, followed by fixation in 4% paraformaldehyde. Immunolocalization was performed using Cy-5, a secondary antibody. After preparing the fluorescent polymyxin B, assays routinely used for fixation and immunolocalization were performed with Lissamine rhodamine-polymyxin B (LRhod-polymyxin B) at a dose of approximately 700 mg/L for 6 hours and with an anti-polymyxin B-monoclonal IgM at a working concentration of approximately 20 mg/L. Cells were also incubated with Oregon Green 514 polymyxin B. LLC-PK1 cells were grown on 35-mm coverslip-bottomed dishes to approximately 40% confluency. After 2 days, solutions of 12.5 μM (n = 4), 75 μM (n = 4) and 375 μM (n = 4) polymyxin B were added to the media. Additionally, LLC-PK1 cells were exposed to 75 μM LRhod-polymyxin B (n = 4) at 4, 8 and 24 hours. The mitochondrial dye used was rhodamine 123 (Rhod 123) at a concentration of 0.1 mg/L. Staining was carried out just prior to imaging each individual dish. Staining involved a 10 min incubation with Rhod 123, followed by two brief washes with PBS (37°C), after which K-P media (37°C) was added. Images were acquired in the confocal mode with simultaneous excitation using a Bio-Rad (Hercules, USA) MRC-1024 combination 2-photon Kr/Ar laser scanning confocal microscope with *trans*-illumination capability on a Nikon Diaphot inverted microscope platform using either a -100 oil immersion objective with a numeric aperture (NA) of 1.4 or a -60 water immersion objective with an NA of 1.2. To avoid the possibility of spectral overlap in the co-localization studies, the emission signals from Texas red and FITC were excited and acquired sequentially using the Kr/Ar laser in single-photon mode. All of the experimental protocols were repeated a total of three times to replicate the findings. The images from the co-localization studies were processed and overlaid using the image processing software Metamorph version 4.0 (Universal Imaging, West Chester, PA). Lateral or *X*-*Z* projections were generated from fields where through-focus optical sections were taken [25, 26].

### Statistical analysis

All quantitative data are expressed as the mean ± SEM. One-factor analyses of variance (ANOVAs) along with confidence intervals around the means and pairwise comparisons were used to analyze the data. Overlapping confidence intervals indicate no significant difference between treatments, which were subsequently confirmed via Kruskal-Wallis tests. Average intensity measurements from the mitochondrial membrane potential experiment were analyzed using Student’s *t*-test. All statistical analyses were performed with GraphPad Prism (version 3.0). Statistical significance was assumed at values of p<0.05.

## Results

### Renal function

Animals exposed to 4 mg/kg/day of polymyxin B for 5 days exhibited no changes in urinary output, but a significant elevation in serum creatinine compared with that of the Saline group (p<0.05) was observed. Thus, creatinine clearance was decreased in the Polymyxin B group (p<0.05, Table 1).

**Table 1:** Overall renal function (mean ± SD) of animals preconditioned with polymyxin B.

Groups	Urinary Output (mL/min)	Urinary Cr (mg/dL)	Serum Cr (mg/dL)	CrCl/100 g (mL/min)
Saline (n = 7)	0.0058±0.0022	76.2±24.0	0.22±0.06	0.70±0.08
PMB (n = 8)	0.0096±0.0091	65.9±32.6	0.52±0.06^a^	0.30±0.33^a^

Cr, creatinine; Cl, clearance; PMB, polymyxin B.

^a^p<0.05 versus the Saline group.

### Renal hemodynamics

Rats in the Polymyxin B group presented a decrease in renal blood flow, while their RVR was increased. All these changes were statistically significant, as shown in Table 2.

**Table 2:** Overall renal hemodynamics (mean ± SD) in animals preconditioned with polymyxin B.

Groups	Heart Rate (bpm)	Blood Pressure (mmHg)	Renal Blood Flow (mL/min)	Renal Vascular Resistance (mmHg/mL/min)
Saline (n = 7)	517±77	100.8±14.6	7.6±1.4	13.6±3.8
PMB (n = 8)	545±61	108.8±11.7	2.3±0.4^a^	47.3±11.0^a^

^a^p<0.05 versus the Saline group.

### Oxidative stress

The effects of polymyxin B treatment on oxidative stress are summarized in Table 3. The Polymyxin B group presented higher levels of oxidative metabolites, urinary peroxides (p<0.05) and TBARS (p<0.05) when compared with the levels in the Saline group. Additionally, catalase activity (p<0.05) and the level of thiols (p<0.05) decreased due to oxidation, which reduced the total antioxidant capacity.

**Table 3:** Indicators of oxidative stress in animals preconditioned with polymyxin B.

Groups	Urinary Peroxides (nmol/g Cr)	Urinary TBARS (nmol/g Cr)	Thiols (nmol/mg protein)	Catalase Activity (nmol/H_2_O_2_ min/mg protein)
Saline (n = 7)	5.2±1.4	38.1±8.9	28.9±6.1	5.4±0.9
PMB (n = 8)	39.1±7.1 ^a^	54.0±12.1^a^	15.7±1.3 ^a^	1.1±0.3 ^a^

Cr, creatinine; TBARS, thiobarbituric acid-reactive substances; PMB, polymyxin B.

^a^p<0.05 versus the Saline group.

### Transmission electron microscope (TEM) results

**Fig 1:**
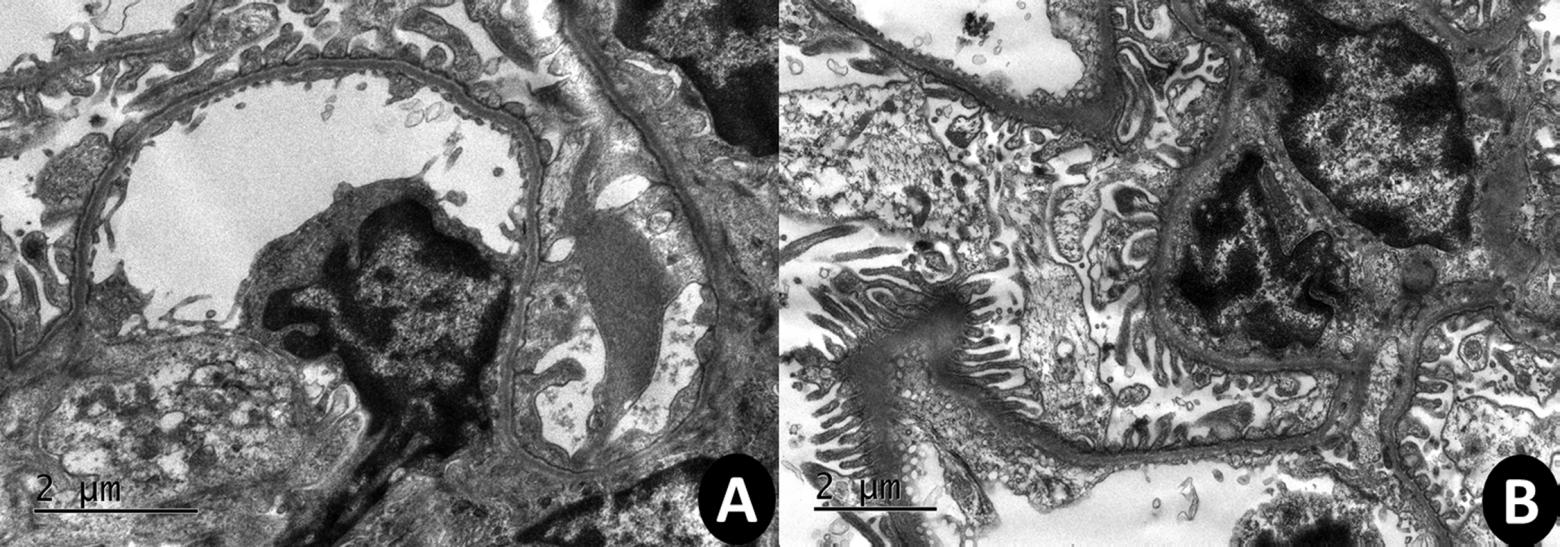
TEM photomicrographs of 2-μm thick kidney sections. A kidney from the Saline group (A) and a kidney from the Polymyxin B group (B). The Polymyxin B group show autophagic vacuoles and mitochondria damage. Images were acquired at 5,000–10,000x magnification.

Electron microscopy revealed specific ultrastructural changes in renal tubular epithelial cells and glomeruli in response to the experimental treatments. Normal tubular epithelial cells, glomeruli cells and their organelles are illustrated in Fig 1A. After exposing rats to polymyxin B, extensive vacuolization, organelle disruption and swollen and disorganized mitochondria were found in tubular epithelial cells (Fig 1B). Additionally, massive podocyte lesions were observed in the glomeruli.

### Western blot detection and quantification of cytochrome c protein in mitochondrial and cytosolic fractions results

**Fig 2:**
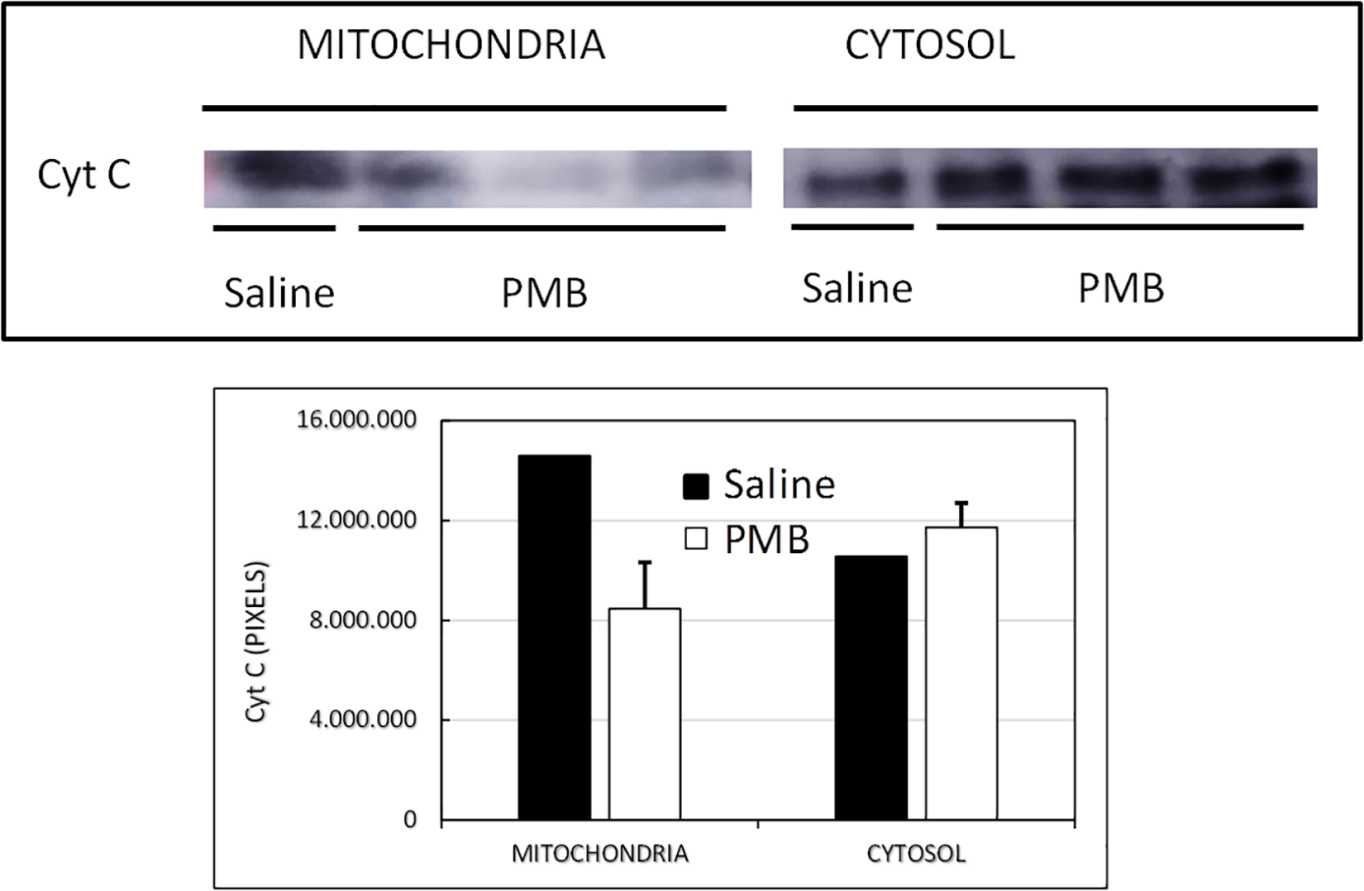
Western blot detection and quantification of cytochrome c protein in mitochondrial and cytosolic fractions of the Saline group and the Polymyxin B group.

Cytochrome c expressions in mitochondrial and cytosolic fractions of kidney cortices were compared. The intensity of the cytochrome c band in the mitochondrial pellet was higher than in cytosolic fraction in the Saline group. Inversely, the animals treated with polymyxin B showed that the cytochrome c band was more intense in the cytosolic fraction than in the mitochondrial pellet of kidney cortices (Fig 2).

### Renal histology

**Fig 3:**
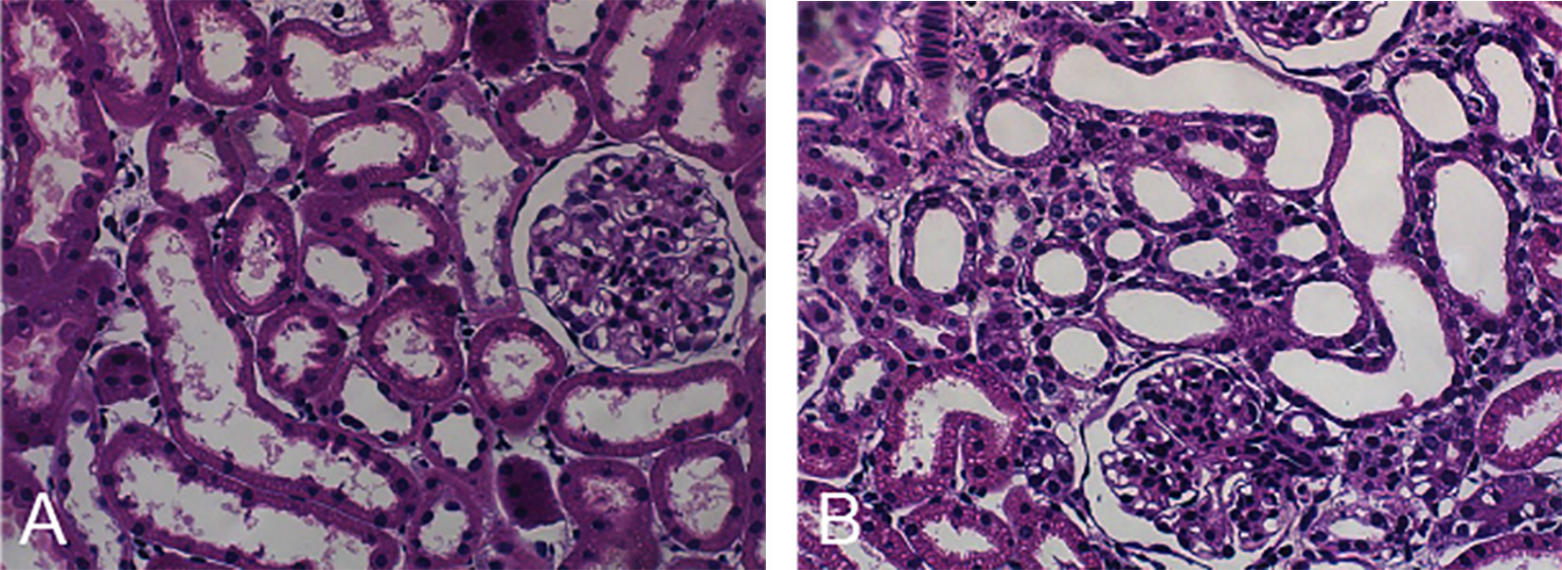
Histological images. Renal tissue from the Saline and Polymyxin B treatment groups. Saline group (A) and Polymyxin B group (B).

Animals treated with polymyxin B showed the presence of tubulointerstitial injury characterized by edema and diffuse inflammatory infiltration of the interstitium, a flattening of tubular cells accompanied by tubular dilatation, focal areas of a denuded basement membrane and tubular necrosis in the renal cortex (Fig 3B).

### Viability and apoptosis in LLC-PK1 cells

**Fig 4:**
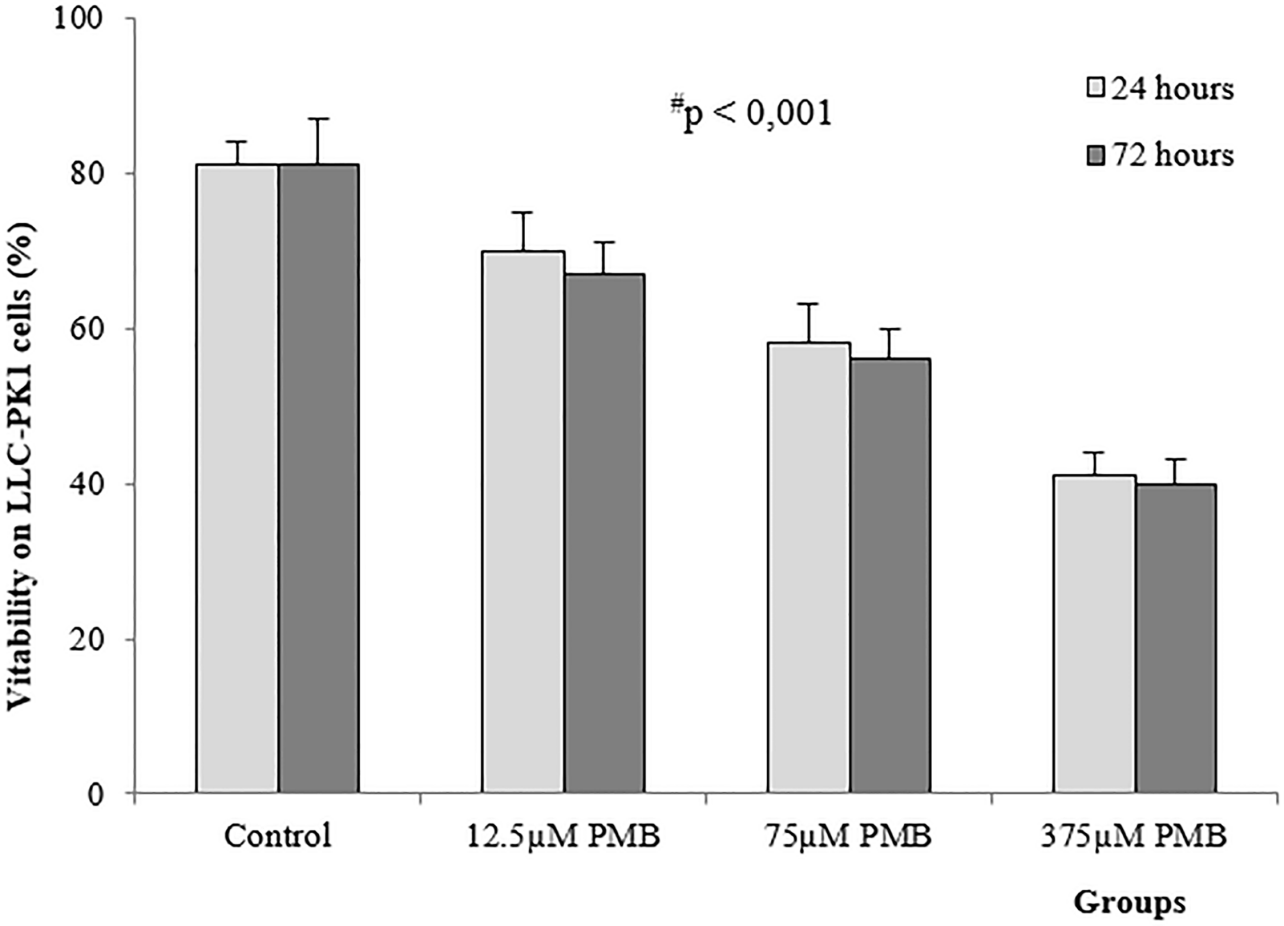
Mean (±SD) viability of LLC-PK1 cells exposed to different concentrations of polymyxin B after 24 and 72 hours (n = 8 in each group). ^#^significantly different from the control group; *significantly different from groups after 24 and 72 hours (p<0.001).

LLC-PK1 cells exposed to polymyxin B at concentrations of 12.5, 75 and 375 μM showed a significant decrease in viability (p<0.001, Fig 4). Toxicity increased when cells were exposed to antibiotics for longer periods of time, as demonstrated by the significant difference between the effects at 24 and 72 hours. The reduced percentage of viable cells in response to 375 μM polymyxin B after 72 hours (41±2%) relative to the effects of other treatments confirms the concentration- and time-dependent toxicity of polymyxin B.

**Fig 5:**
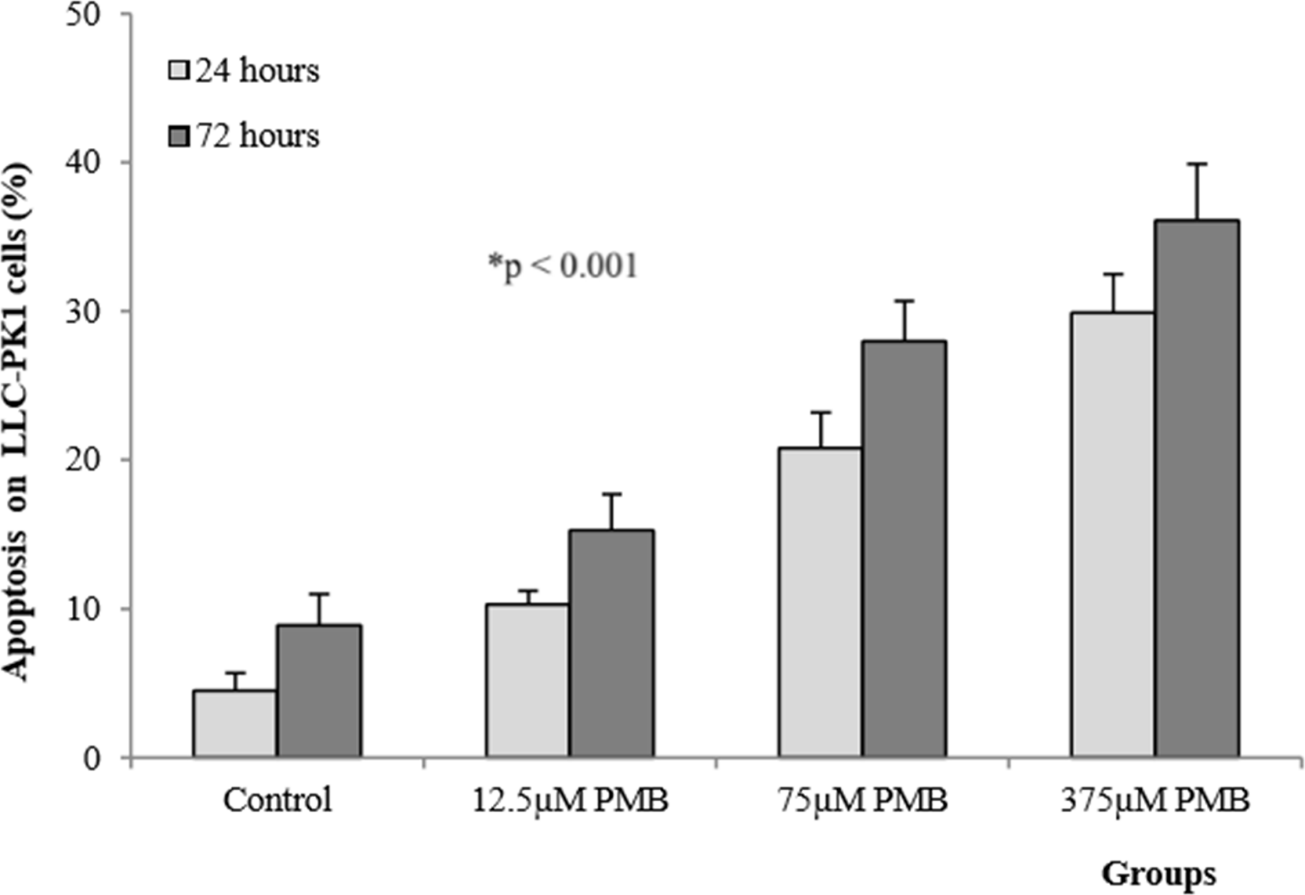
Mean (±SD) level of apoptosis in LLC-PK1 cells exposed to different concentrations of polymyxin B after 24 and 72 hours (n = 8 in each group). *significant difference relative to the control group and after 24 and 72 hours (p<0.001).

As shown in Fig 5, increased polymyxin B concentrations increased the population of apoptotic cells after 24 and 72 hours (p<0.001). Multiple comparisons of the number of apoptotic cells between the groups revealed significant differences after 24 and 72 hours (p<0.001). These findings demonstrate a concentration- and time-dependent toxic effect on cells after the administration of polymyxin B. Accordingly, a larger number of apoptotic cells was observed following treatment with 375 μM polymyxin B after 72 hours (36.1±3.8%) that were observed under the other conditions.

### LDH and nitric oxide levels

Table 4 shows that LLC-PK1 cells exposed to 375 μM polymyxin B had increased LDH levels at 24 and 72 hours (p<0.01). The significantly higher levels of this cellular membrane damage mediator at 72 hours than at 24 hours indicate a time-dependent cytotoxicity of polymyxin B. However, nitrite concentrations were increased only at 72 hours.

**Table 4:** Membrane damage mediator levels in LLC-PK1 cells exposed to 375 μM polymyxin B concentration.

	LDH (%)		NO (μM/mg protein)	
Groups	24 hours	72 hours	24 hours	72 hours
Control	2.2±0.1	1.8±0.2	0.18±0.05	0.63±0.10
PMB	13.8±1.9^a^	22.7±1.8^b^^c^	0.22±0.04	1.47±0.35^b^^c^

LDH, lactate dehydrogenase; NO, nitric oxide; PMB, polymyxin B.

^a^p<0.001 versus the control group at 24 hours

^b^p<0.001 versus the control group at 72 hours and

^c^p<0.01 versus the PMB group at 24 hours.

### The polymyxin B immunolocalization in LLC-PK1 cells using Lissamine rhodamine-polymyxin B

**Fig 6:**
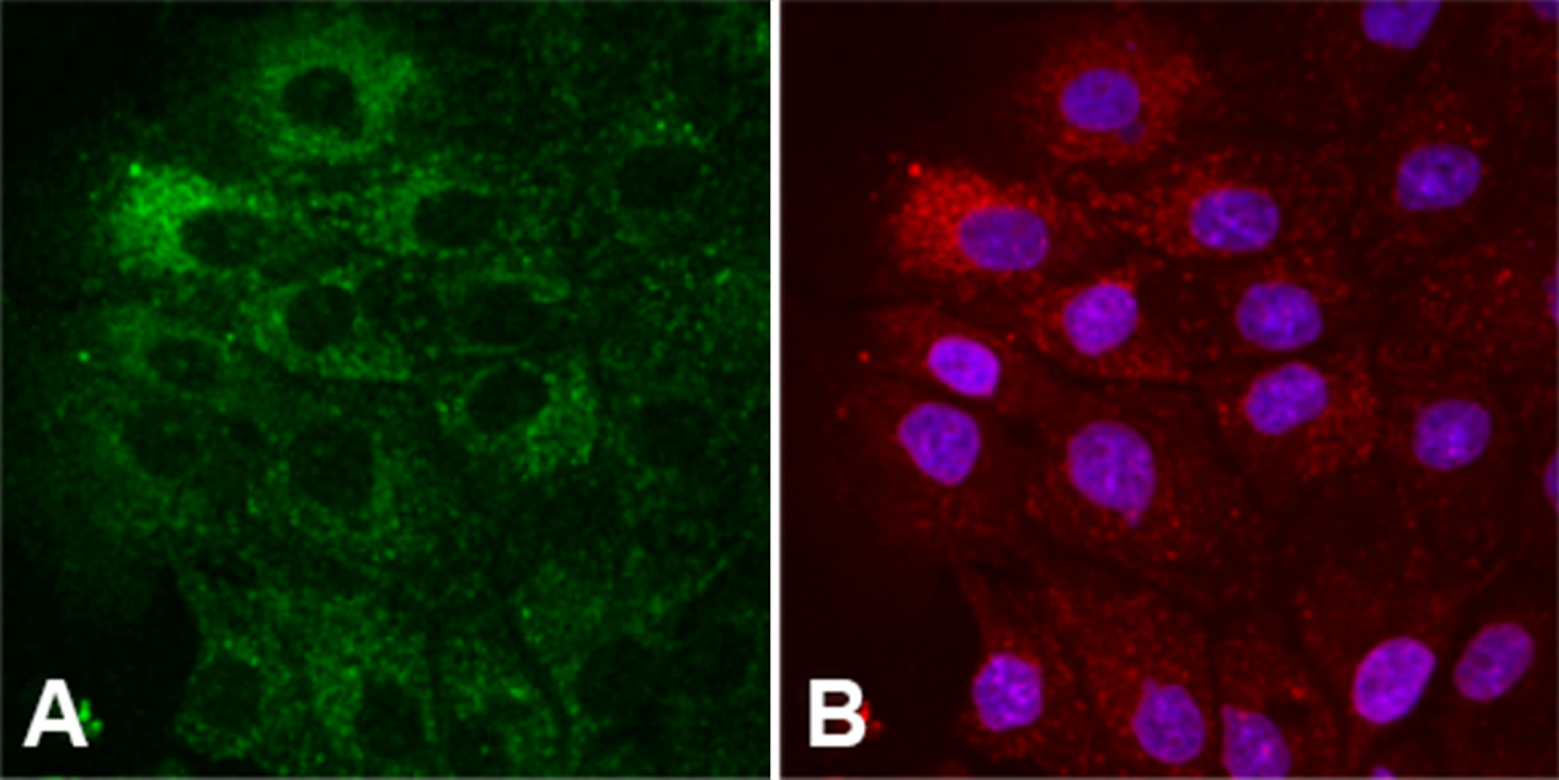
Structural images of LLC-PK1 cells. Polymyxin B localization in LLC-PK1 cells cultivated with 700 mg/L LRhod-polymyxin B after 6 hours of incubation (A) and immunolocalization with 20 mg/L anti-polymyxin B-monoclonal IgM (B).

Polymyxin B localization in LLC-PK1 cells is shown in Fig 6. The autofluorescence signal in the green range represents LLC-PK1 cells exposed to LRhod-polymyxin B (Fig 6A). LRhod-polymyxin B and anti-polymyxin B antibodies show different staining patterns, including a punctate-vesicular pattern, diffuse cytosolic staining with a greatly reduced nucleolar staining, and possibly mitochondrial localization, in LLC-PK1 cells after 6 hours of incubation (Fig 6B).

**Fig 7:**
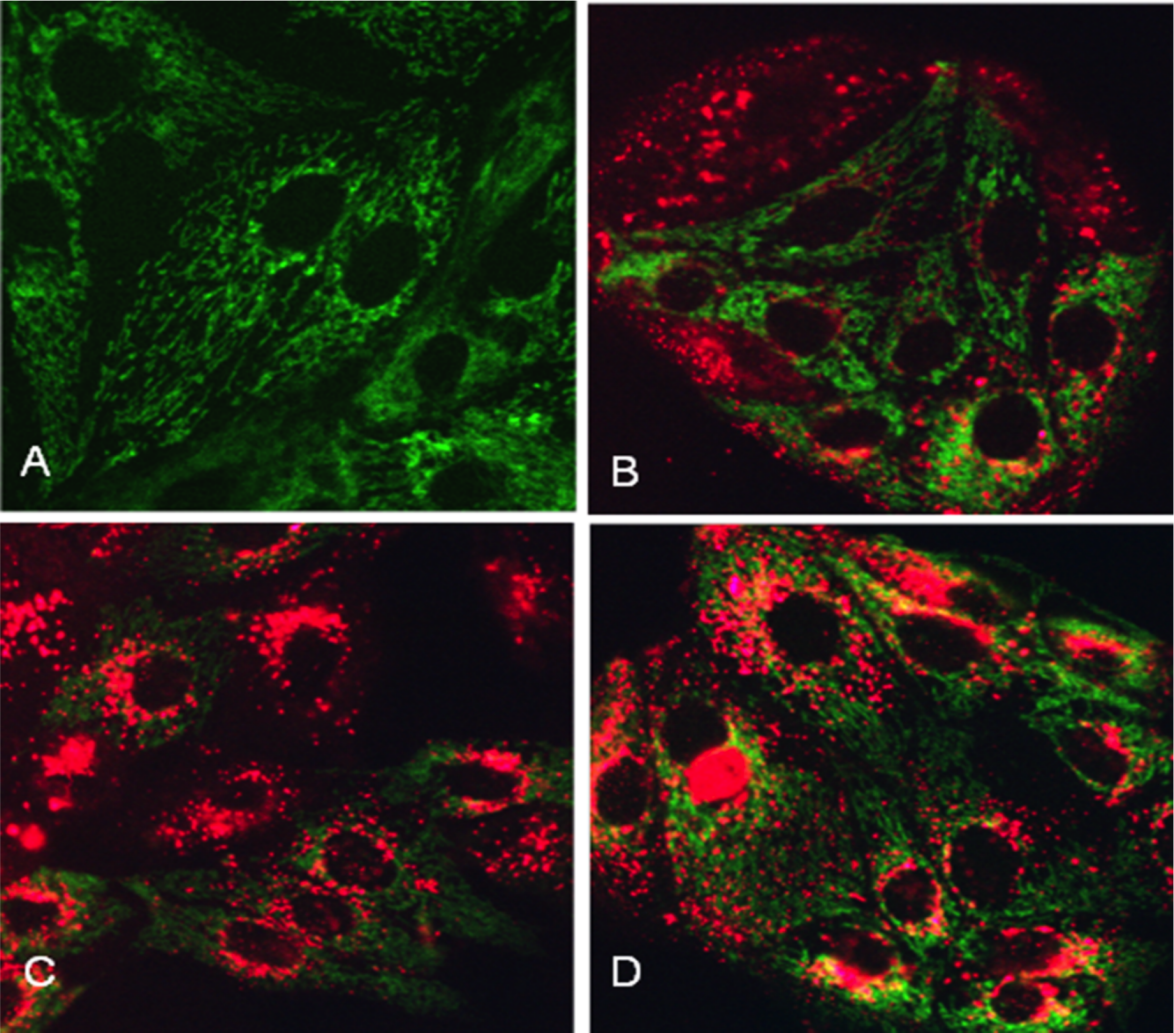
Images of LLC-PK1 cells incubated with Lissamine rhodamine-polymyxin B (LRhod-polymyxin B). Control cells incubated in physiological media (A), cells exposed to 75 μM LRhod-polymyxin B for 4 hours (B), cells exposed to 75 μM LRhod-PMB for 8 hours (C), and cells exposed to 75 μM LRhod-polymyxin B for 24 hours (D).

LLC-PK1 cells present a diffuse pattern of green fluorescence (Fig 7A). In contrast, LLC-PK1 cells exposed to 75 μM LRhod-polymyxin B show a decreased fluorescence intensity in the mitochondria at 4, 8 and 24 hours (Fig 7B, 7C and 7D). Rhod 123 is a dye that accumulates in the mitochondria based on membrane potential [25].

## Discussion

Polymyxin B and colistin (polymyxin E) are nephrotoxic and can promote renal injury. These polymyxin antimicrobials have a very narrow therapeutic window due to the necessary balance between their antibacterial activity and nephrotoxicity [3, 27]. Our toxicity data suggest that polymyxin B can induce AKI by interfering with total renal function, redox status, cellular integrity and. mitochondrial membrane. These results, which present a panoramic view encompassing organ and cellular damage, exemplify the paradigm of translational science for toxic kidney diseases.

We demonstrated that the administration of polymyxin B in rats increases serum creatinine levels and decreases creatinine clearance. Abrupt declines in glomerular filtration rate and increases in serum creatinine levels have been confirmed in polymyxin B-induced AKI in humans and rats [8, 12, 28, 29]. Additionally, alterations to renal hemodynamics following polymyxin B administration act as a key regulator of microvasculature function leading to vasoconstriction. In our study, high RVR was associated with vasoconstriction, which is a critical component of the acute toxic effect of polymyxin B on the kidneys, leading to reduced intrarenal perfusion and intracapillary hydraulic pressure, thereby, decreasing the glomerular filtration rate. Thus, these changes to renal hemodynamics can sensitize the proximal tubule cells, resulting in endothelial dysfunction associated with increases in oxidative metabolites and decreases in antioxidant enzymes (catalase and glutathione peroxidase) in the kidney.

Oxidative stress has been shown to play a crucial role in the nephrotoxicity caused by polymyxins [30, 31, 32]. Our data show that the administration of 4 mg/kg/day of polymyxin B in rats results in elevated ROS generation, as demonstrated by increases in urinary peroxides and TBARS, and in a decrease in antioxidant capacity, as demonstrated by reduced thiol levels and catalase activity in renal tissue.

The kidneys metabolize and excrete several xenobiotics, and a high renal blood flow increases drug delivery, which results in high concentrations of toxins in the renal medulla and interstitium [33]. Renal toxicity occurs due to the extensive cellular uptake of potential toxins and drugs by both apical and basolateral transport systems. These toxins or drugs induce destabilization in intracellular membranes and alterations in membrane permeability, which leads to pathological ROS generation inside or outside the cell [33, 34]. High levels of ROS, or oxidants, (for example: hydrogen peroxides, hydroxyl radicals and hypochlorous acid) interfere with signaling cascades and result in harmful effects on various biological molecules and structures, which can cause oxidative injury via nucleic acid oxidation, protein damage and lipid peroxidation [33, 34, 35]. Concomitantly, excessive amounts of ROS decrease the activities of antioxidant enzymes that regulate the redox status of the cell, inducing cell death [33, 35, 36]. Several authors have shown that the administration of free radical scavengers, or antioxidants, such as ascorbic acid, n-acetylcysteine, and melatonin, has beneficial effects against polymyxin-induced nephrotoxicity via the amelioration of oxidative injury [30, 31, 32].

This study analyzed data on redox imbalances in the presence of polymyxin B to evaluate the role of mitochondria in nephrotoxic injuries once its dynamics have been profoundly altered. Mitochondrial fragmentation may relate to the excess generation of ROS, which increases the susceptibility to stimuli that initiate the entry of proapoptotic proteins. Mitochondria have been reported to serve as a link between apoptosis and autophagy, with increasing levels of stress resulting in increased caspase activating, apoptosis and, finally, necrotic cell death. We observed alterations to morphological characteristics of cellular ultrastructure, such as extensive vacuolization, organelle disruption and swollen and disorganized mitochondria, in tubular epithelial cells. In addition, the glomeruli cells in treated animals displayed massive podocyte lesions [37, 38].

Mitochondrial membrane injury releases cytochrome c in the cytosol. Cytosolic cytochrome c is associated with processing of apoptosis and cell lysis during necrosis and cell injury [39, 40]. We showed elevation of cytosolic cytochrome c in polymyxin B treated group, which confirms that the cell damage is due to mitochondrial dysfunction. Cystosolic cytochrome c activates caspase-dependent apoptosis and the apoptosis-inducing factor that initiates the caspase independent pathway of apoptosis by causing DNA fragmentation and chromatin condensation [39].

Histological studies of kidney tissues from patients with toxic AKI have been shown to have interstitial edema and tubular degenerative changes, with luminal ectasia, a loss of brush borders, cytoplasmic simplification and vacuolization, nuclear enlargement and pleomorphism, cellular necrosis, and the presence of apoptotic figures [32, 41]. In our study, we observed edema and inflammatory infiltration in the interstitium, a flattening of tubular cells accompanied by tubular dilatation, denuded basement membranes, and tubular necrosis in the renal cortex of kidney tissues of rats treated with polymyxin B. Yun and colleagues demonstrated that kidney tissues with polymyxin B-induced nephrotoxicity present tubular dilatation and degeneration in the cortex. In addition, polymyxin B in the kidney tissues of mice was observed to be distributed in the renal cortex and, specifically, in the renal LLC-PK1 cells used as a model for proximal tubular cells [42].

Polymyxin B-induced nephrotoxicity has been demonstrated to involve cellular uptake at the apical membrane in LLC-PK1 cells [43]. Renal tubular cells are vulnerable to injury due to nephrotoxicity once polymyxin B achieves a high affinity to renal tissue, which results in its potential for nephrotoxicity [6, 29, 44]. LLC-PK1 cells show similarities with human proximal tubule cells, including their membrane transport activities, membrane enzymes and microvilli [45]. In addition, polymyxin B uptake into LLC-PK1 cells occurs via endocytosis at the apical membrane [43]. As a result of this uptake, polymyxin B accumulates within renal tubular cells, leading to several forms of cellular injury or death [33, 43].

Apoptosis occurs as a defense mechanism in a variety of disease states or in the presence of toxic drugs [40, 46]. Our results show that the presence of escalating concentrations of polymyxin B (12.5, 75 and 375 μM) in LLC-PK1 cells results in decreased cell viability and a greater number of apoptotic cells over time, demonstrating the concentration- and time-dependent cytotoxicity of polymyxin B. This concentration- and time-dependent nephrotoxicity has been previously described in animals and humans based on damage to tubular epithelial cells [1, 29, 47]. Nephrotoxicity has also been associated with higher cumulative doses during systemic polymyxin B or colistin therapy in clinical practice [5, 28, 48]. In vitro and in vivo studies have described similar results for the concentration- and time-dependent nephrotoxicity induced by polymyxin B [6, 29, 47].

In this study, we demonstrated an increase in the levels membrane damage mediators, as shown by increased LDH levels and the generation of signaling NO species in LLC-PK1 cells exposed to 375 μM polymyxin B. The plasma membrane is an important site of damage in AKI, and once the membrane is damaged, increased levels of NO can act in synergy to unbalance the redox status of the cell. Reactive species such as NO cross cellular membranes, promote oxidative changes and interfere with several signaling cascades, resulting in harmful effects on biological molecules and structures [35, 49].

Under physiological conditions, nitric oxide synthase (NOS) produces a significant amount of NO involved in the regulation of mitochondrial respiration and other cellular regulatory functions. NO, particularly that induced by inducible NOS, can cause oxidative cellular injury when produced in large amounts. This NO can inhibit the electron transport chain (ECT) and can increase the formation of superoxides (strong oxidants) [49]. Additionally, superoxides and NO participate in secondary reactions to generate more reactive species, which can induce mitochondrial dysfunction, resulting in decreased ATP formation, cell death and organ failure [35]. In addition to these effects, the excessive or aberrant generation of ROS and the presence of micromolar concentrations of NO also affect inner mitochondrial membranes, which permits the movement of proapoptotic proteins such as caspases and Bcl-2 family members through the membrane [50]. The increase in proteolytic activity caused by caspases induces an irreversible process in cells undergoing apoptosis, which involves chromatin condensation, DNA fragmentation, protein degradation and the formation of apoptotic bodies that are phagocytosed by macrophages or adjacent normal cells [40, 46].

The treatment of LLC-PK1 cells with polymyxin B demonstrated different staining patterns, such as punctate-vesicular, diffuse cytosolic staining but with a greatly reduced nucleolar staining, and possible mitochondrial localization, in these cells following 6 hours of incubation with 700 mg/L LRhod-polymyxin B and an anti-polymyxin B antibody and with 75 μM LRhod-polymyxin B and Rhod-123 for 4, 8 and 24 hours, as illustrated by decreases in fluorescence intensity. Rhod-123 is a dye that is taken up into tubular cells and is localized in the mitochondria, followed by its accumulation in active mitochondria [50], and our results suggest that LLC-PK1 cells exposed to polymyxin B have altered mitochondria function caused by changes in mitochondrial membrane polarization. Mitochondria in the kidney have a several intracellular functions. This organelle used 85–90% of the oxygen in the cells to generate energy in the form of ATP and to produce small amounts of ROS that provide a delicate balance between the generation of ROS and the function of the antioxidant system to control the cellular redox status and to promote the modulation of cell death pathways [34]. Recent studies have reported that mitochondrial dysfunction plays an important role in the mechanism of drug-induced AKI [10, 51]. Azad et al have demonstrated that polymyxin B induces mitochondrial morphological fragmentation and the loss of the mitochondrial membrane potential, which resulted in concentration- and time-dependent mitochondrial dysfunction in rat kidney proximal tubular cells (NRK-52E) [10]. A concentration- and time-dependent toxicity in LLC-PK1 cells induced by polymyxin B was also observed in our study.

Despite the insights presented here, further studies on the interaction between the nephrotoxic effects of polymyxin B on the metabolic functions of the kidney and its effects on cellular structural integrity must be conducted.

Our results show decreased glomerular filtration rate and tubulointerstitial injury, along with mitochondrial dysfunction, which modifies the redox status in LLC-PK1 cells. Large increases in the production of signaling species (NO, hydrogen peroxides) and ROS result in cell death via apoptosis and are associated with the concentration- and time-dependent cytotoxicity of polymyxin B.

In conclusion, polymyxin B accumulates to a high degree in the kidneys, corroborating previous results. Our data highlight the links between the toxic effects of polymyxin B as part of a new understanding of translational science as it applies to renal diseases.

## References

[1] Zavascki AP, Goldani LZ, Li J, Nation RL. Polymyxin B for the treatment of multidrug-resistant pathogens: a critical review. J Antimicrob Chemother. 2007; 60:1206–15. 17878146 10.1093/jac/dkm357

[2] Kasiakou SK, Michalopoulos A, Soteriades ES, Samonis G, Sermanaides GJ, Falagas ME. Combination therapy with intravenous colistin for management of infections due to multidrug-resistant gram-negative bacteria in patients without cystic fibrosis. Antimicrob Agents Chemother. 2005; 49:3136–46. 16048915 10.1128/AAC.49.8.3136-3146.2005 PMC1196256

[3] Velkov T, Roberts KD, Nation RL, Thompson PE, Li J. Pharmacology of polymyxins: new insights into an 'old' class of antibiotics. Future Microbiol. 2013; 8:711–24. 10.2217/fmb.13.39 23701329 PMC3852176

[4] Kvitko CH, Rigatto MH, Moro AL, Zavascki AP. Polymyxin B versus other antimicrobials for the treatment of pseudomonas aeruginosa bacteraemia. J Antimicrob Chemother. 2011; 66:175–9. 10.1093/jac/dkq390 20961911

[5] Kubin CJ, Ellman TM, Phadke V, Haynes LJ, Calfee DP, Yin MT. Incidence and predictors of acute kidney injury associated with intravenous polymyxin B therapy. J Infect. 2012; 65:80–7. 10.1016/j.jinf.2012.01.015 22326553

[6] Abdelraouf K, Braggs KH, Yin T, Truong LD, Hu M, Tam VH. Characterization of polymyxin B-induced nephrotoxicity: implications for dosing regimen design. Antimicrob Agents Chemother. 2012; 56:4625–9. 10.1128/AAC.00280-12 22687519 PMC3421883

[7] Schetz M, Dasta J, Goldstein S, Golper T. Drug-induced acute kidney injury. Curr Opin Crit Care. 2005; 11:555–65. 16292059 10.1097/01.ccx.0000184300.68383.95

[8] Arnold TM, Forrest GN, Messmer KJ. Polymyxin antibiotics for gram-negative infections. Am J Heath Syst Pharm. 2007; 64:819–26. 10.2146/ajhp060473 17420197

[9] Falagas ME, Kasiakou SF. Nephrotoxicity of intravenous colistin; a prospective evaluation. Crit Care. 2006; 10:R27 16507149

[10] Azad MA, Akter J, Rogers KL, Nation RL, Velkov T, Li J. Major pathways of polymyxin-induced apoptosis in rat kidney proximal tubular cells. Antimicrob Agents Chemother. 2015; 59:2136–43. 10.1128/AAC.04869-14 25624331 PMC4356794

[11] Danner RL, Joiner KA, Rubin M, Patterson WH, Johnson N, Ayers KM, et al Purification, toxicity, and antiendotoxin activity of polymyxin B nonapeptide. Antimicrob Agents Chemother. 1989; 33:1428–34. 2554795 10.1128/aac.33.9.1428 PMC172678

[12] Fonseca CD, Watanabe M, Vattimo MFF. Role of heme oxygenase-1 in polymyxin B-induced nephrotoxicity in rats. Antimicrob Agents Chemother. 2012; 56:5082–7. 10.1128/AAC.00925-12 22802257 PMC3457368

[13] Fernandes SM, Martins DM, da Fonseca CD, Watanabe M, Vattimo M de F. Impact of iodinated contrast on renal function and renal hemodynamics in rats with chronic hyperglycemia and chronic kidney disease. Biomed Res Int. 2016; 2016:3019410 10.1155/2016/3019410 27034930 PMC4789389

[14] American Veterinary Medical Association. AVMA guidelines on euthanasia, 2013 update. Available: http://www.avma.org/issues/animal_welfare/euthanasia.pdf. Accessed 01 Feb 2016.

[15] Dorea EL, Yu L, De Castro I, Campos SB, Ori M, Vaccari EM, et al Nephrotoxicity of amphotericin B is attenuated by solubilizing with lipid emulsion. J Am Soc Nephrol. 1997; 8:1415–22. 9294833 10.1681/ASN.V891415

[16] Halliwell B, Long LH, Yee TP, Lim S, Kelly R. Establishing biomarkers of oxidative stress: the measurement of hydrogen peroxide in human urine. Curr Med Chem. 2004; 11:1085–92. 15134507 10.2174/0929867043365404

[17] Akerboom TPM, Sies H. Assay glutathione, glutathione disulfide, and glutathione mixed disulfides in biological samples. Methods Enzymol. 1981; 77:373–82. 7329314 10.1016/s0076-6879(81)77050-2

[18] Aebi H. Catalase in vitro. Methods Enzymol. 1984; 105:121–6. 6727660 10.1016/s0076-6879(84)05016-3

[19] Beuge JA, Aust SD. The thiobarbituric acid assay. Methods Enzymol. 1978, 52:306–7.

[20] Borges AA, El-Batah PN, Yamashita LF, Santana Ados S, Lopes AC, Freymuller-Haapalainen E, et al Neuroprotective effect of oral choline administration after global brain ischemia in rats. Nutr Neurosci. 2015; 18:265–74. 10.1179/1476830514Y.0000000125 24754536

[21] Borutaite V, Budriunaite A, Morkuniene R, Brown GC. Release of mitochondrial cytochrome c and activation of cytosolic caspases induced by myocardial ischaemia. Biochim Biophys Acta. 2001; 1537:101–9. 11566253 10.1016/s0925-4439(01)00062-x

[22] Shih W, Hines WH, Neilson EG. Effects of cyclosporin A on the development of immune-mediated interstitial nephritis. Kidney Int. 1988; 33:1113–8. 3261370 10.1038/ki.1988.119

[23] Filatov MV, Varfolomeeva EY. Active dissociation of Hoechst 33342 from DNA in living mammalian cells. Mutat Res. 1995; 327: 209–215. 7870089 10.1016/0027-5107(94)00189-c

[24] Green LC, Wagner DA, Glogowki J, Skipper PL, Wishnok JS, Tannenbaum SR. Analysis of nitrate, nitrite, and [15 N] nitrate in biological fluids. Anal Biochem. 1982; 126:131–8. 7181105 10.1016/0003-2697(82)90118-x

[25] Molitoris BA, Sandoval RM. Pharmacophotonics: utilizing multi-photon microscopy to quantify drug delivery and intracellular trafficking in the kidney. Adv Drug Deliv Rev. 2006; 58:809–23. 17064810 10.1016/j.addr.2006.07.017

[26] Yu W, Sandoval RM, Molitoris BA. Rapid determination of renal filtration function using an optical ratiometric imaging approach. Am J Physiol Renal Physiol. 2007; 292:F1873–80. 17311910 10.1152/ajprenal.00218.2006

[27] Velkov T, Thompson PE, Nation RL, Li J. Structure-activity relationships of polymyxin antibiotics. J Med Chem. 2010; 53:1898–916. 10.1021/jm900999h 19874036 PMC2907661

[28] Akajagbor DS, Wilson SL, Shere-Wolfe KD, Dakum P, Charurat ME, Gilliam BL. Higher incidence of acute kidney injury with intravenous colistimethate sodium compared with polymyxin B in critically ill patients at a tertiary care medical center. Clin Infect Dis. 2013; 57:1300–3. 10.1093/cid/cit453 23840000

[29] Abdelraouf K, He J, Ledesma KR, Hu M, Tam VH. Pharmacokinetics and renal disposition of polymyxin B in an animal model. Antimicrob Agents Chemother. 2012; 56:5724–7. 10.1128/AAC.01333-12 22908162 PMC3486600

[30] Yousef JM, Chen G, Hill PA, Nation RL, Li J. Ascorbic acid protects against the nephrotoxicity and apoptosis caused by colistin and affects its pharmacokinetics. J Antimicrob Chemother. 2012; 67:452–9. 10.1093/jac/dkr483 22127588 PMC3254197

[31] Ozyilmaz E, Ebinc FA, Derici U, Gualbahar O, Goktas G, Elmas C, et al Could nephrotoxicity due to colistin be ameliorated with the use of N-acetylcysteine? Intensive Care Med. 2011; 37:141–6. 10.1007/s00134-010-2038-7 20845026

[32] Yousef JM, Chen G, Hill PA, Nation RL, Li J. Melatonin attenuates colistin-induced nephrotoxicity in rats. Antimicrob Agents Chemother. 2011; 55:4044–9. 10.1128/AAC.00328-11 21709095 PMC3165279

[33] Perazella MA. Renal vulnerability to drug toxicity. Clin J Am Soc Nephrol. 2009; 4:1275–83. 10.2215/CJN.02050309 19520747

[34] Murphy MP. How mitochondria produce reactive oxygen species. Biochem J. 2009; 417:1–13. 10.1042/BJ20081386 19061483 PMC2605959

[35] Andrades MÉ, Morina A, Spasić S, Spasojević I. Bench-to-bedside review: sepsis—from the redox point of view. Crit Care. 2011; 15:230 10.1186/cc10334 21996422 PMC3334726

[36] Jassem W, Heaton ND. The role of mitochondria in ischemia/reperfusion injury in organ transplantation. Kidney Int. 2004; 66:514–7. 15253700 10.1111/j.1523-1755.2004.761_9.x

[37] Agarwal A, Dong Z, Harris, Murray P, Parikh SM, Rosner MH, et al; Acute Dialysis Quality Initiative XIII Working Group. Cellular and molecular mechanisms of acute kidney injury. J Am Soc Nephrol. 2016; 27:1288–99. 10.1681/ASN.2015070740 26860342 PMC4849836

[38] Johnson-Lyles DN, Peifley K, Lockett S, Neun BW, Hansen M, Clogston J, et al Fullerenol cytotoxicity in kidney cells is associated with cytoskeleton disruption, autophagic vacuole accumulation, and mitochondrial dysfunction. Toxicol Appl Pharmacol. 2010; 248:249–58. 10.1016/j.taap.2010.08.008 20713077 PMC2949473

[39] Small DM, Gobe GC. Cytochrome c: potential as a noninvasive biomarker of drug-induced acute kidney injury. Expert Opin Drug Metab Toxicol. 2012; 8:655–64. 10.1517/17425255.2012.679657 22475359

[40] Havasi A, Borkan SC. Apoptosis and acute kidney injury. Kidney Int. 2011; 80:29–40. 10.1038/ki.2011.120 21562469 PMC4625984

[41] Markowitz GS, Perazella MA. Drug-induced renal failure: a focus on tubulointerstitial disease. Clin Chim Acta. 2005; 351:31–47. 15563870 10.1016/j.cccn.2004.09.005

[42] Yun B, Azad MA, Wang J, Nation RL, Thompson PE, Roberts KD, et al Imaging the distribution of polymyxins in the kidney. J Antimicrob Chemother. 2015; 70:827–9. 10.1093/jac/dku441 25377569 PMC4319485

[43] Abdelraouf K, Chang KT, Yin T, Hu M, Tam VH. Uptake of polymyxin B into renal cells. Antimicrob Agents Chemother. 2014; 58:4200–2. 10.1128/AAC.02557-14 24733472 PMC4068554

[44] Bergen PJ, Landersdorfer CB, Zhang J, Zhao M, Lee HJ, Nation RL, et al Pharmacokinetics and pharmacodynamics of ‘old’ polymyxins: what is new? Diagn Microbiol Infect Dis. 2012; 74:213–23. 10.1016/j.diagmicrobio.2012.07.010 22959816 PMC3477253

[45] Nielsen R, Birn H, Moestrup SK, Nielsen M, Verroust P, Christense EI. Characterization of a kidney proximal tubule cell line, LLC-PK1, expressing endocytotic active megalin. J Am Soc Nephrol. 1998; 9:1767–76. 9773777 10.1681/ASN.V9101767

[46] Elmore S. Apoptosis: a review of programmed cell death. Toxicol Pathol. 2007; 35:495–516. 17562483 10.1080/01926230701320337 PMC2117903

[47] Azad MA, Finnin BA, Poudyal A, Davis K, Li J, Hill PA, et al Polymyxin B induces apoptosis in kidney proximal tubular cells. Antimicrob Agents Chemother. 2013; 57:4329–35. 23796937 10.1128/AAC.02587-12 PMC3754291

[48] Pogue JM, Lee J, Marchaim D, Yee V, Zhao JJ, Chopra T, et al Incidence of and risk factors for colistin-associated nephrotoxicity in a large academic health system. Clin Infect Dis. 2011; 53:879–84 10.1093/cid/cir611 21900484

[49] Moncada S, Erusalimsky JD. Does nitric oxide modulate mitochondrial energy generation and apoptosis? Nat Rev Mol Cell Biol. 2002; 3:214–20. 11994742 10.1038/nrm762

[50] Plotnikov EY, Kazachenko AV, Vyssokikh MY, Vasileva AK, Tcvirkun DV, Isaev NK, et al The role of mitochondria in oxidative and nitrosative stress during ischemia/reperfusion in the rat kidney. Kidney Int. 2007; 72:1493–502. 17914353 10.1038/sj.ki.5002568

[51] Hall AM, Rhodes GJ, Sandoval RM, Corridon PR, Molitoris BA. In vivo multiphoton imaging of mitochondrial structure and function during acute kidney injury. Kidney Int. 2013; 83:72–83. 10.1038/ki.2012.328 22992467 PMC4136483

